# Revisit incidence of complications after impacted mandibular third molar extraction: A nationwide population-based cohort study

**DOI:** 10.1371/journal.pone.0246625

**Published:** 2021-02-22

**Authors:** Ya-Wei Chen, Lin-Yang Chi, Oscar Kuang-Sheng Lee

**Affiliations:** 1 School of Dental Medicine, University of Pennsylvania, Philadelphia, PA, United States of America; 2 Institute of Clinical Medicine, National Yang-Ming University, Taipei, Taiwan; 3 Taipei City Hospital, Taipei, Taiwan; 4 Stem Cell Research Center, National Yang-Ming University, Taipei, Taiwan; 5 Department of Medical Research, Taipei Veterans General Hospital, Taipei, Taiwan; 6 Department of Orthopedics and Traumatology, Taipei Veterans General Hospital, Taipei, Taiwan; Thamar University, Faculty of Dentistry, YEMEN

## Abstract

Most of complications after impacted mandibular third molar (iLM3) extraction surgeries are transient and resolved spontaneously within one or two weeks, but some of them are more complicated and required further treatments to alleviate the symptoms. The aim of study is to revisit incidence and predictors of complications after iLM3 surgery by reviewing previous literature and investigating a population-based data. From Taiwan National Health Insurance Research Database, records of 16,609 patients who had received iLM3 extraction under ambulatory settings were retrieved for analysis. Outcomes of interest included dry socket (DS), prolonged temporomandibular joint symptoms (TMD), and surgical site infection (SSI), which necessitated additional appointments to manage. Odds ratios of having those complications between different variables were analyzed. The incidence rates of DS, TMD, and SSI were 3.6%, 0.41%, 0.17%, respectively; while they ranged from 0.33–19.14% (DS), 0–4.17% (TMD), and 0.2–5.17% (SSI) in previous studies. Logistic regression revealed DS significantly correlated with complexity of odontectomy (2.5-fold of risk) and history of gingivitis or pericoronitis (1.3-fold of risk). More TMD was found in female than male patients (0.5% versus 0.3%). However, no factors associated with SSI was found; neither did we find aging as a risk in association with any of above complications. Compared to previous studies, our data supports that surgical intervention should be considered in iLM3 with risk of gingivitis or pericoronitis to reduce the occurrence of DS. The original information in this article, which provides a “real-world” evidence, along with the organizing data we summarized from previous article, can serve as a reference for clinicians in assessing the complication risks before treatment of iLM3.

## Introduction

Third molar is the most commonly seen impacted tooth in the mouth, with a higher occurrence rate in the lower jaw than the upper jaw [1]. To surgically extract symptom-free or pathology-free impacted third molars as a preventive manner has always been a debate between clinicians for a long time [2, 3]. In the past decades, evidence has shown an increased incidence of periodontal breakdown or other dental morbidities on the adjacent second molars when third molars were present or impacted; the prevalence rises as the patient ages [4–8]. In the new White Paper released by the American Association of Oral and Maxillofacial Surgeons (AAOMS) in 2017, they advocated third molars that are associated with disease or at higher risk of developing disease should be surgically extracted [9]. Suggestions have also been made to surgically remove asymptomatic or pathology-free impacted third molars prior to the development of pathology at the time when the post-surgical healing is optimal and with a lower risk of complications [10].

Weighing the risks and costs associated with impacted third molar surgery is an important part for both patients and clinicians. Before any surgical procedures, the patient should be provided with full information on the pros and cons of surgery, as well as perioperative risks and postoperative complications. Surgical removal of iLM3 often necessitates odontectomy, which is a procedure of gingival flap elevation and tooth sectioning, to take the tooth piece-by-piece out of bone. Therefore, it is very common to have postoperative inflammatory symptoms such as pain, swelling, and trismus after the surgery, which is transient and resolves spontaneously within two weeks. However, some postoperative complications are more severe, and the symptoms and conditions do not resolve without additional management. For example, prolonged temporomandibular joint symptoms after iLM3 surgery might happen in some patients, even though the facial swellings are resolved. This is usually due to too much lateral forces having been executed during extraction, which causes disc displacement or traumatic inflammation around the joint complex. Dry socket (a.k.a. alveolar osteitis) is a delayed healing, inflammatory complication on the extraction wound due to loss of blood clot. The symptoms of dry socket include bad odor from the mouth, dull throbbing pain and/ or referred pain to the ear or other teeth but without classic signs of infection. Obviously, patients worry more about the complications which prompt them for additional dental visit and treatment [11]. These are, therefore, the complications of interest we will be looking at in this study.

Reviewing recent literature, a very wide range of complication rates from impacted third molar extraction have been reported (4.6% to 30.9%), which stemmed from diverse definitions of complications, different study designs, and settings [12–15]. For example, one study has shown the overall complication rate of 4.6%, which is reported from a sample of 583 patients having maxillary or mandibular third molar extraction by one single oral maxillofacial surgeon in the U.S. Their reported postoperative complication rate per tooth was 3.4%, while these complications included dry socket, persistent oroantral communication, infection, hematoma, bone spicules, pain, or swelling [14]. Another prospective study reported 6.9% incidence of dry socket, infection, and paresthesia of inferior alveolar nerve from a total of 550 impacted mandibular third molars (iLM3) at a single private dental practice in Canada [15]. To our knowledge, none of the previous articles have been conducted on the incidence of complications based on population dataset, and very few studies have investigated a prolonged symptoms on temporomandibular joint after iLM3 extraction. Therefore, the aim of present study is to use nationwide, population-based database to investigate the incidence and risk predictors of surgical site infection (SSI), dry socket (DS), and prolonged temporomandibular joint symptoms (TMD), which resulted in patient’s additional appointment for further treatment. This article also aims to provide a concise review of the incidence of these complications, so a comparison of data can be made easily based on different chronological, nationality, and settings.

## Materials and methods

One million randomly sampled population data from Taiwan Nationwide Health Insurance Research Database (NHIRD) was used in this retrospective cohort study. The Taiwan National Health Insurance (NHI) Program is a mandatory nationwide single-payer social health insurance system that has been operating since 1995. It offers comprehensive medical and dental care coverage to 99.9% of all 23 million people [16]. The NHI program allows beneficiaries to receive dental disease prevention and treatment including iLM3 surgical extraction, as well as full mouth prophylactic periodontal scaling twice a year.

The NHIRD has the complete NHI claims registries. All personal identification information is encrypted. Therefore, the consent forms from subjects were waived. The study protocol was reviewed and approved by the IRB at Taipei City Hospital, Taipei, Taiwan (TCHIRB- 10808004-E). As an observational study, it conformed to the STROBE (Strengthening the Reporting of Observational Studies in Epidemiology) guidelines [17]. Cases were identified based on the *International Classification of Disease*, *9th Edition*, *Clinical Modification (ICD-9-CM)* diagnostic code.

Patients aged 16 to 55 years old who had received iLM3 surgical extraction under ambulatory settings from Jan. 1 to Dec. 31, 2012 were identified by a combination of diagnostic codes (*ICD-9-CM* 520.6) and the NHI procedure codes (92015c and 92016c). Both 92015c and 92016c are coded for flap-elevated surgical extractions. The code 92015c defines a simple or non-complicated odontectomy for soft tissue impaction or mesial-tilted partial bony impaction. The code 92016c defines a more complicated odontectomy for total bony impaction or horizontal bony impaction. The latter procedure often requires a larger flap elevation and more bone removal to facilitate tooth segmentation and removal. Individuals with preexisting diagnosis of temporomandibular joint diseases (TMD) (*ICD-9-CM* 524.60, 524.62, 524.63, or 524.69) were excluded, so the identified postoperative diagnosis related to TMD can be referred as post-extraction complications. Those who had missing data were also excluded.

All study participants were followed from postoperative one week to six months. Any symptoms or diagnoses within one week after the surgeries were considered as physiological or transient postoperative complications, which were not our outcomes of interest, therefore not retrieved. The outcome variables were the occurrence of postoperative SSI, DS, and TMD. The onset of the following complications was validated as the patients had at least ≥ 1 postoperative outpatient or inpatient visit with the following diagnostic codes. For postoperative TMD, it was defined by the TMD diagnostic codes (*ICD-9-CM* 524.60, 524.62, 524.63, or 524.69), which were made by physicians or dentists based on patient’s medical history and chief complaints, clinical, and radiographic examinations. Postoperative infection was defined as having a diagnostic code of abscess or cellulitis (*ICD-9-CM* 528.3, 682.0), while DS was defined as having the diagnostic code for dry socket (*ICD-9-CM* 526.5).

The risk factors included demographic variables, including age (categorized into four groups), gender; complexity of surgery (92015c and 92016c); pathologic variables, including history of abscess or cellulitis around iLM3 and history of gingivitis or pericoronitis (*ICD-9-CM* 523.0, 523.1) on iLM3 within 180 days before surgical intervention; and the condition of oral hygiene maintenance within three years preoperatively. The different state of oral hygiene maintenance is based on patients’ frequency of prophylactic full mouth ultrasonic dental scaling (FMUS) (91004c). We defined good oral hygiene maintenance if the patients received FMUS more than three times within three years preoperatively; fair oral hygiene maintenance if two to three times of FMUS within three years; and poor if never or only one time of FMUS.

Descriptive analyses were computed for investigated variables. Chi-square test was used to test the difference in investigated variables between patients with and without postoperative complications. Multivariate logistic regression model was applied to estimate relative risk of groups of patients with complications, expressed as adjusted odds ratios (AOR) and with respective 95% confident intervals (95% CI). Microsoft SQL Server 2015 (Microsoft Corp, Redmond, WA, USA) was used to collect and manage data, while all computing and statistical analyses were carried out using SAS v. 9.4 (SAS Institute Inc., Cary, NC, USA). *P* < 0.05, 2-sided was used as a threshold for statistical significance.

## Results

A total number of 16,609 patients who had one or two iLM3 extracted over a year were retrieved. If a patient had two iLM3 extracted within the same year, only the first one was chosen for the analysis. Therefore, the sample size of iLM3 was 16,609 in total. There were more female patients than male patients who had iLM3 extracted (F: M = 54%: 46%). Nearly half of the patients were in the age group of 16–25, only 7% of patients were in the age group of 46–55. Only 235 patients (1.41%) had prior abscess or cellulitis localized around iLM3. But 75% of the patients had gingivitis or pericoronitis before iLM3 extraction. All descriptive statistics is shown in Table 1.

**Table 1:** Demographic features of study subjects (N = 16,609).

Characteristics		%	n
**Gender**			
	Female	54.01	8,971
	Male	45.99	7,638
**Age, year**			
	16–25	49.62	8,241
	26–35	32.99	5,479
	36–45	10.31	1,712
	46–55	7.09	1,177
**Complexity of odontectomy**			
	Non-complicated	32.63	5,419
	Complicated	60.29	11,190
**History of abscess or cellulitis**			
No		98.59	16,374
Yes		1.41	235
**History of gingivitis or pericoronitis**			
	No	24.84	4,126
	Yes	75.16	12,483
**State of oral hygiene maintenance**			
	Good	25.19	4,183
	Fair	52.95	8,794
	Poor	21.87	3,632

**Fig 1:**
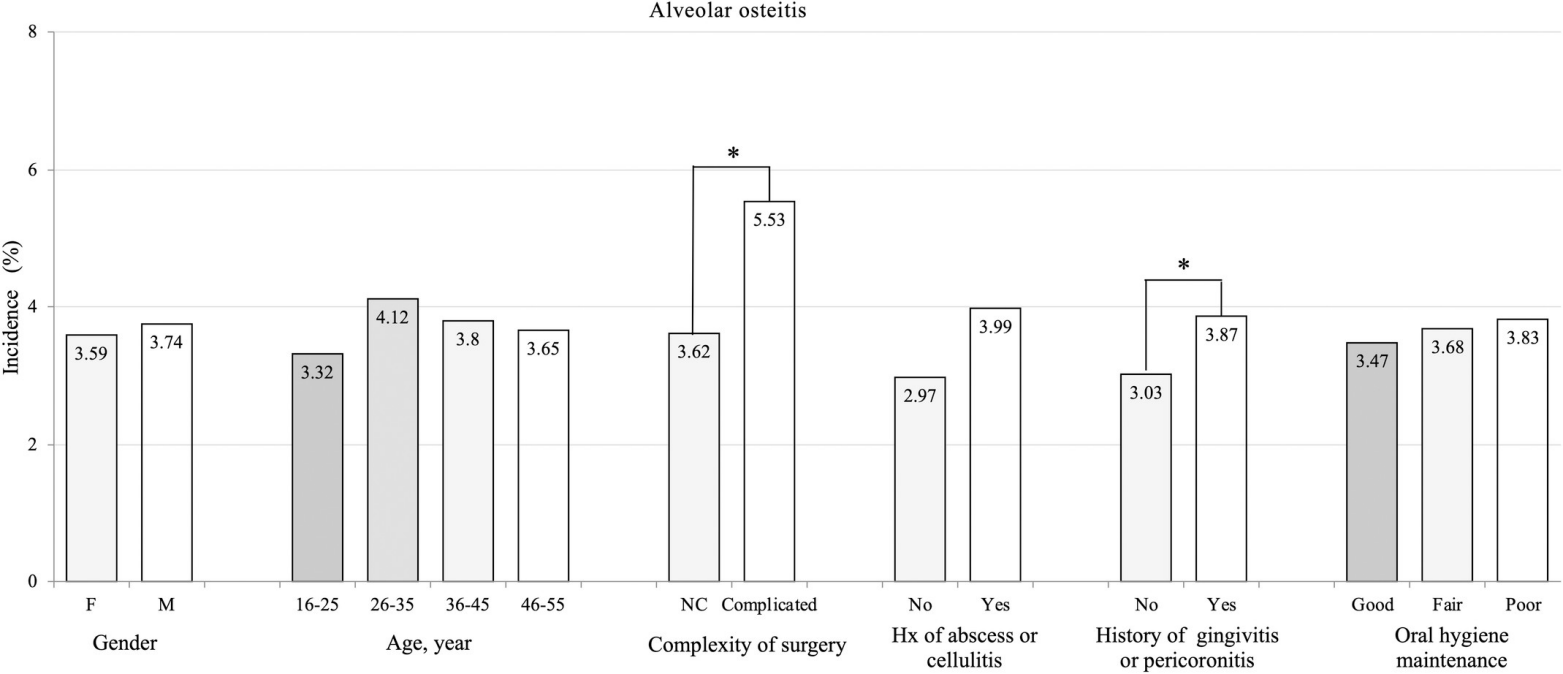
Incidence of Dry Socket (DS) in different variables.

Univariate analyses between the study variable and each of the complications is shown in Table 2. The overall cumulative complication rate for iLM3 extraction was 4.2%. The incidence of DS was the highest (3.66%), while the incidence of SSI was very low (0.17%). About 4 out of 1000 patients had temporomandibular joint symptoms and sought for treatment (0.41%) within 6 months of observation following surgical extraction. The incidence rates of DS in different subgroups can be found in Fig 1. Univariate analyses showed that complexity of surgery (*p* = 0.0008) and history of gingivitis or pericoronitis (*p* = 0.0128) were associated with DS. Although the incidence of DS in patients with a history of abscess or cellulitis was higher (5.53%) than those without history of abscess or cellulitis (3.63%), there was no statistical difference. In terms of TMD, female patients had a significantly higher incidence than male patients (0.5% versus 0.3%, respectively) (P = 0.0437). Although a higher incidence of TMD in complicated odontectomy (0.46%) than in non-complicated odontectomy (0.3%), no statistical significance was found. None of our study variables were related to SSI.

**Table 2:** Study variables grouped by complications.

Variables	SSI					DS					TMD				
	Yes		No		*p*	Yes		No		*p*	Yes		No		*p*
	(n = 29)		(n = 16,580)		value	(n = 608)		(n = 16001)		value	(n = 68)		(n = 16,541)		value
	n	%	n	%		n	%	n	%		n	%	n	%	
**Gender**															
Female	19	65.52	8952	53.99	0.2134	322	52.96	8649	54.05	0.5958	45	66.18	8926	53.96	0.0437^b^
Male	10	34.48	7628	46.01		286	47.04	7352	45.95		23	33.82	7615	46.04	
**Age, year**															
16–25	15	51.72	8226	49.61	0.9453	274	45.07	7967	49.79	0.6892	32	47.06	8209	49.63	0.6892
26–35	10	34.48	5469	32.99		226	37.17	5253	32.83		21	30.88	5458	33.00	
36–45	2	6.90	1710	10.31		65	10.69	1647	10.29		10	14.71	1702	10.29	
46–55	2	6.90	1175	7.09		43	7.07	1134	7.09		5	7.35	1172	7.09	
**Complexity of odontectomy**															
Non-complicated	6	20.69	5413	32.65	0.1983	161	26.48	5258	32.86	0.0008^b^	17	25.00	5402	32.66	0.1789
Complicated	23	79.31	11167	67.35		447	73.52	10743	67.14		51	75.00	11139	67.34	
**History of abscess or cellulitis**															
No	28	96.55	16346	98.59	0.3387^a^	595	97.86	15779	98.61	0.1239	66	97.06	16308	98.59	0.2502^a^
Yes	1	3.45	234	1.41		13	2.14	222	1.34		2	2.94	233	1.41	
**History of gingivitis or pericoronitis**															
No	8	27.59	4118	24.84	0.7321	125	20.57	4001	25.01	0.0128^b^	17	25.00	4109	24.84	0.9759
Yes	21	72.41	12462	75.16		483	79.43	12000	74.99		51	75.00	12432	75.16	
**State of oral hygiene maintenance**															
Good	6	20.69	4177	25.19	0.4797	145	23.85	4038	25.24	0.6884	18	26.47	4165	25.18	0.5204
Fair	14	48.28	8780	52.96		324	53.29	8470	52.93		39	57.35	8755	52.93	
Poor	9	31.03	3623	21.85		139	22.86	3493	21.83		11	16.18	3621	21.89	

DS, dry socket. TMD, prolonged temporomandibular joint symptoms, SSI, surgical site infection.

^a^ Fisher exact test

^b^ statistical significance.

**Fig 2:**
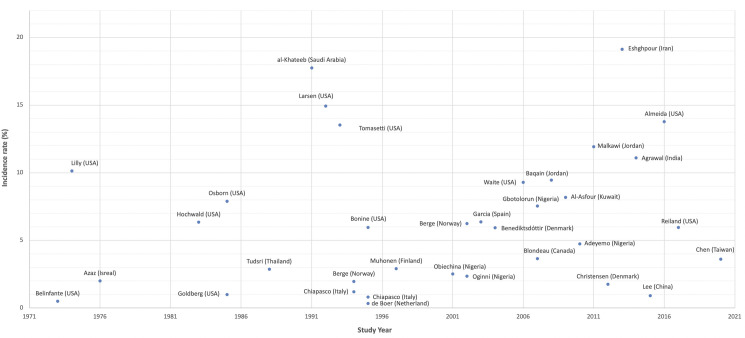
Graph demonstrating incidence of Dry Socket (DS) after iLM3 extraction chronologically in different studies and setting.

**Fig 3:**
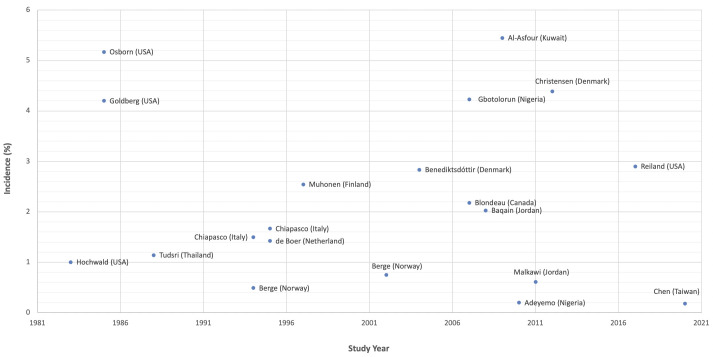
Graph demonstrating incidence of surgical site infection after iLM3 extraction chronologically in different studies and setting.

To compare our results to previous literature, we have found 531 relevant studies from PubMed, which addressed complications after impacted third molar extraction. Longitudinal cohort articles with data of DS, SSI, TMD incidence were summarized and tabulated (S1–S3 Tables). Under different study settings, the range of sample size in each study was from 114 to 11,255. The DS incidence was 0.33 to 19.14%, SSI incidence was 0.20 to 5.17%, and TMD incidence was 0 to 4.17%. The chorological overviews of the DS and SSI complication rates are shown in Figs 2 and 3.

**Fig 4:**
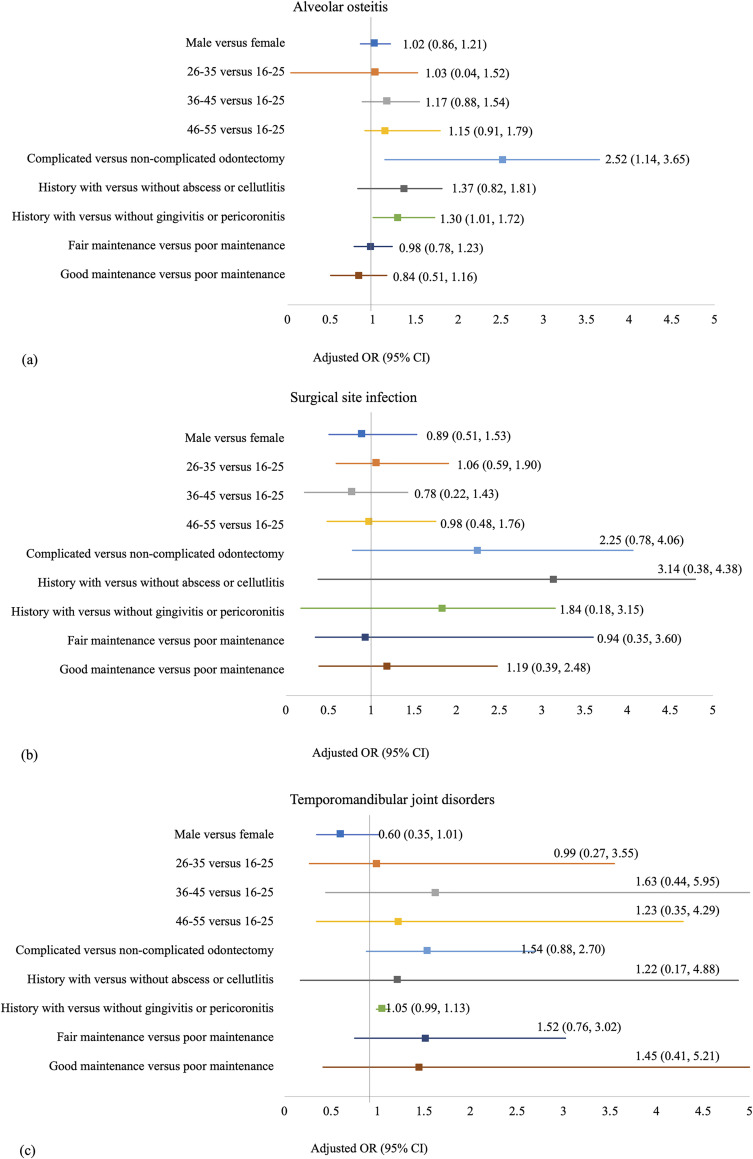
Factors related to the complications in multivariate regression model. The forest plot displays the adjusted odds ratio and 95% confidence interval of (a) dry socket, (b) temporomandibular joint disorders, and (c) surgical site infection.

Multivariate analyses revealed that patients who received complicated odontectomy had significantly higher risk of DS (Fig 4A). The risks were more than 2.5-fold compared to the patients who received non-complicated odontectomy (95% CI = 1.14, 3.65; *p* = 0.0010). The AOR for the history of gingivitis or pericoronitis was 1.30 (95% CI = 1.01, 1.72; *p* = 0.0482), suggesting that participants with a history of gingivitis or pericoronitis was 30% more likely to have DS than those without the history. However, none of the factors showed statistically significant association with the SSI or TMD (Fig 4B and 4C).

## Discussion

Dry socket (DS) is the most frequent complication after dental extraction and one of the most studied subjects in the field of oral surgery [18]. The characteristic symptom of DS is increasing postoperative pain surrounding the extraction wound with the onset of 2 to 4 days after dental surgery, accompanying with the signs of intra-alveolar blood clot disintegration and yellow-gray necrotic tissue layer covering the surface [19, 20]. Excessive trauma from extraction, aggressive curettage on the wound, and bacteria invasion have all been reported to predispose local fibrinolytic activity and become the etiology of dry sockets [21, 22]. In our review of literature, there were 33 articles had data of DS incidence after iLM3 extraction. As seen in Fig 2 and S1 Table, a wide range of DS complication rate after iLM3 extraction is shown. But half of the studies showed less than 6% of incidence, and three-fourth of studies had less than 10% of incidence. Geographically, most of the studies (20/34) were conducted in America and Europe, six studies were conducted in middle east countries, four studies in Africa, and three studies in Asia.

For the factors that might contribute to the occurrence of DS, difficulty of extractions, age, female gender, smoking, pre-existing infections, and oral hygiene have all been cited [12, 18, 23–26]. However, we found the most influencing factor associated with DS is the complexity of surgery and followed by the history of gingivitis or pericoronitis. But no difference of incidence found between different age or gender groups, or between different states of oral hygiene maintenance. These findings are very similar to Osborn’s study in 1985, which had a comparable sample size to ours. In their study, they found the highest incidence of DS with complete bony impaction (10.1%), followed by partial bony impaction (7.6%), soft tissue impaction (3.7%), and erupted lower third molars (2.0%). Although no statistical analysis was conducted in their article, their data also failed to show older patients had more complication rates than younger ones [12].

Our study revealed that 75% of patient population had a history of gingivitis or pericoronitis on iLM3, and 1.41% of patients with history of abscess or cellulitis around iLM3 within 180 days before iLM3 extraction. However, only the history of gingivitis or pericoronitis was associated with an elevated DS frequency, but the history of abscess or cellulitis was not. We speculate the reason is that when a patient is experiencing abscess or cellulitis in the mouth, the clinicians would mostly postpone impacted tooth extraction until the acute infectious condition is controlled. However, if the patient is having gingivitis or pericoronitis, which is apparently a very minor and limited site condition, extraction of impacted tooth would still be performed. Hence, the impact of pre-existing infections can still be considered a predisposing factor. Because the extraction is not performed for the existing abscess or cellulitis condition, the association between DS and history of abscess/ cellulitis cannot be found in this study.

The incidence of SSI of iLM3 in this study was quite low (0.17%) compared to other studies (S2 Table). We consider the substantial difference in these numbers is from the lack of tangible definition of postoperative infection and inflammation. Both of the conditions have symptoms of erythema, swelling, heat, and pain. However, postoperative inflammation resolves gradually with time and does not show the signs of presence of bacteria, while SSI has persistent or progression of inflammation due to bacterial invasion, concomitant with or without purulent discharge or fever. Our criteria of postoperative SSI were only confined to more severe infectious condition—abscess or cellulitis, which was stricter than other studies, therefore resulting in a lower frequency compared to other studies.

TMD is another outcome of interest in our study, which has not been widely discussed until recently. After surgical extraction of iLM3, it is not uncommon to see symptoms of TMJ pain, clicking, or limitation of movement of lower jaw [27–29]. Extraction of iLM3 as a causative factor of TMJ pain is plausible, for it requires a prolonged wide opening of the jaw, as well as a considerable lateral force applied to the mandible when executing extraction [30]. Previous studies have shown the positive association of between third molar extraction and the occurrence of TMD [27, 30–34]. In a retrospective matched-paired cohort study conducted in the U.S., the authors found a relative risk for TMD in patients who had third molar extraction was 1.4 compared to individuals who did not have third molar extraction, with the TMD incidence in patients with third-molar removal was 0.7%, while in subjects without third-molar removal was 0.5% [33]. Another study, which conducted prospectively with questionnaires, has found a much higher incidence rate (34.3 per 100 person-year) of TMD in patients who had undergone third molar removal, when compared to individuals who had not (8.8 per 100 person-year) [34]. In the current study, we found the incidence of TMD to be 0.4% after iLM3 extraction. We also found more female patients experiencing TMD than male patients, and TMD happened more in more complex surgical procedures. These findings were similar to the findings in other studies [30, 33, 36, 37].

Chronic TMJ symptoms (more than 3 to 6-month duration) after third molar surgery were found to be relatively rare according to previous studies [35, 37]. Our study looked at the development of TMD symptoms within 6 months after iLM3 surgery, but we did not investigate the duration of TMD. Nevertheless, a prospective, controlled study has found most of the TMJ symptoms after iLM3 surgery to be more of acute status. They found decrease range of jaw opening and difficulties chewing hard food were found only up to 1 month after surgery. Significant increased pain intensity was noted 1 week after surgery (in 56% of patients), while the pain intensity was decreasing and lasted up to 6 months postoperatively (in 13% of patients) [35].

During postoperative period, other uncontrollable causative factors, such as parafunction, trauma secondary to other long dental procedures, or psychological stress, etc., might potentially encounter, and direct causal-effect of iLM3 extraction and TMD cannot be made. However, from the current study and previous literature, a tendency for temporary and acute TMD symptoms after third molar extraction can be expected in 0.04–4.17% of incidence, this possible sequela shouldn’t be neglected.

Another interesting finding in our research is that none of the postoperative complications were found to be correlated to the age of patients, although some studies have pointed out a significant increase in surgical morbidity and postoperative complications as patients became older [38–40]. We speculate the reason might be due to the “real-world” case selection in older patient group—when most local dental clinicians encountered older or systemically-compromised patients, they choose to treat easier cases and refer difficult cases to more experienced clinicians or specialists. It has been proved that clinical experience is inversely related to the incidence of postoperative complications [41], therefore, there might be a cancellation effect from it. Interestingly, our point-of-view concurs with those rigorous studies on TMD complications in third molar surgeries. Those studies have found the risk of TMD is not increased greatly in older age groups. They stated the reason that third molar removal is not likely to be a large risk factor for TMD in older population risk of TMD is because the simpler extraction patterns observed in older people. [14, 33, 42] Further rigorous investigations will be needed to elucidate the correlation of age and post-iLM3 extraction complications.

The strength of this study is the application of a population-based data, which enables us to trace rare complications after surgeries. Compared to the hospital or dental clinic-based data, population-based data minimizes selection bias and provides real-world evidence. There are, however, some fundamental limitations when conducting a retrospective study with the NHIRD. First, as a retrospective study, the natural limitation is that some potential confounding factors can attribute to biases and cannot be obtained from the claims database. Second, although the patients can access to most of dental treatment under the national insurance coverage, they might not utilize the service. Thus, the claims insurance data might underestimate disease incidence or prevalence. Third, another important postoperative complication, paresthesia, should also be investigated but could not be done because many diagnoses for untreatable symptoms or conditions are often omitted from the medical record since medication is not prescribed or treatments are not performed.

In conclusion, the present study provides a summarized complication rates of previous literature and the first population-based evidence regarding the incidence and predictors of SSI, DS, and TMD after iLM3 extraction. We believe data based on this large-scale dataset can carry out unbiased evaluation of correlation between the variables and outcomes. The current study showed complexity of surgery and history of gingivitis or pericoronitis are the two independent risk predictors associated with DS. Hence, prophylactic surgical extraction of iLM3 before the occurrence of gingivitis or pericoronitis is encouraged. However, our results do not find older age patients have higher risks of these complications.

## Supporting information

S1 Table Summarized incidence (%) of alveolar osteitis after iLM3 surgical extraction. (*incidence calculated based on patient number, not impacted tooth number).
(DOCX)

S2 Table Summarized incidence of (%) of surgical site infection after iLM3 surgical extraction. (*incidence calculated based on patient number, not impacted tooth number).
(DOCX)

S3 Table Summarized incidence of (%) of prolonged temporomandibular joint disorders after iLM3 surgical extraction. (DOCX)
