# Supplementary material for: Revisit incidence of complications after impacted mandibular third molar extraction: A nationwide population-based cohort study
Source: PLoS One. 2021 Feb 22;16(2):e0246625. doi: 10.1371/journal.pone.0246625 (PMC7899344; doi:10.1371/journal.pone.0246625)
Supplement: S1 Table — (*incidence calculated based on patient number, not impacted tooth number). (DOCX) [file pone.0246625.s001.docx]

S1 Table.

| **Year** | **Author** | **Country** | **Type of study** | **Setting** | **Numbers of iLM3** | **Incidence (%)** |
| --- | --- | --- | --- | --- | --- | --- |
| 1973 | Belinfante | USA | Prospective cohort | Private clinics (Multi-center) | 400 | 0.50 |
| 1974 | Lilly | USA | Retrospective cohort | Hospitals  (Multi-center) | 1836 | 10.13 |
| 1976 | Azaz | Israel | Prospective cohort | University-Medical center | 200 | 2.00 |
| 1983 | Hochwald | USA | Retrospective cohort | University | 598 | 6.35 |
| 1985 | Goldberg | USA | Retrospective cohort | Private clinic, hospital  (Multi-center) | 500 | 1.00 |
| 1985 | Osborn | USA | Prospective cohort | Private clinics | 11255 | 7.90 |
| 1988 | Tudsri | Thailand | Prospective cohort | Unknown | 175  (patient) | 2.86* |
| 1991 | al-Khateeb | Saudi Arabia | Retrospective cohort | University-hospital | 363 | 17.76 |
| 1992 | Larsen | USA | Prospective cohort | University | 134 | 14.93 |
| 1993 | Tomasetti | USA | Retrospective cohort | Private clinic | 3244 | 13.53 |
| 1994 | Berge | Norway | Prospective cohort | University | 204 | 1.96 |
| 1995 | de Boer | Netherland | Retrospective cohort | University-hospital | 2390 | 0.33 |
| 1995 | Chiapasco | Italy | Retrospective cohort | University-hospital | 1500 | 0.80 |
| 1995 | Bonine | USA | Prospective cohort | Private clinic | 654 | 5.96 |
| 1997 | Muhonen | Finland | Retrospective cohort | University | 550 | 2.91 |
| 2001 | Obiechina | Nigeria | Retrospective cohort | University-hospital, clinics  (Multi-center) | 717 | 2.51 |
| 2002 | Oginni | Nigeria | Retrospective cohort | University-hospital | 127 | 2.36 |
| 2002 | Berge | Norway | Prospective cohort | University | 1458  (patients) | 6.24* |
| 2003 | Garcia | Spain | Prospective cohort | University-hospital | 267 | 6.37 |
| 2004 | Benediktsdóttir | Denmark | Prospective cohort | University | 388 | 5.93 |
| 2006 | Waite | USA | Prospective cohort | University-hospital clinic | 635 | 9.29 |
| 2007 | Blondeau | Canada | Prospective cohort | Private clinic | 550 | 3.64 |
| 2007 | Gbotolorun | Nigeria | Prospective cohort | University-hospital | 331 | 7.55 |
| 2008 | Baqain | Jordan | Prospective cohort | University-hospital | 148  (patients) | 9.46* |
| 2009 | Al-Asfour | Kuwait | Retrospective | University | 110 | 8.18 |
| 2010 | Adeyemo | Nigeria | Retrospective cohort | University-hospital | 506 | 4.74 |
| 2011 | Malkawi | Jordan | Prospective cohort | University-hospital | 327  (patients) | 11.93* |
| 2012 | Christensen | Denmark | Retrospective cohort | University | 114 | 1.75 |
| 2013 | Eshghpour | Iran | Cross-sectional study | Private clinic | 256 | 19.14 |
| 2014 | Agrawal | India | Retrospective cohort | University, hospital (Multi-center) | 270 | 11.11 |
| 2015 | Lee | China | Prospective cohort | University | 546 | 0.92 |
| 2016 | Almeida | USA | Retrospective cohort | University | 363 | 13.77 |
| 2017 | Reiland | USA | Retrospective cohort | Private clinic | 1895 | 5.96 |
| 2020 | Chen | Taiwan | Retrospective cohort | National database | 16609 | 3.66 |
