# Supplementary material for: Revisit incidence of complications after impacted mandibular third molar extraction: A nationwide population-based cohort study
Source: PLoS One. 2021 Feb 22;16(2):e0246625. doi: 10.1371/journal.pone.0246625 (PMC7899344; doi:10.1371/journal.pone.0246625)
Supplement: S2 Table — (*incidence calculated based on patient number, not impacted tooth number). (DOCX) [file pone.0246625.s002.docx]

S2 Table.

| **Year** | **Author** | **Country** | **Type of study** | **Setting** | **Numbers of iLM3** | **Incidence**  **(%)** |
| --- | --- | --- | --- | --- | --- | --- |
| 1983 | Hochwald | USA | Retrospective cohort | University | 598 | 1.00 |
| 1985 | Goldberg | USA | Retrospective cohort | Private clinic, hospital  (Multi-center) | 500 | 4.20 |
| 1985 | Osborn | USA | Prospective cohort | Private clinics | 11255 | 5.17 |
| 1988 | Tudsri | Thailand | Prospective cohort | Unknown | 175  (patients) | 1.14* |
| 1994 | Berge | Norway | Prospective cohort | University | 204 | 0.49 |
| 1995 | de Boer | Netherland | Retrospective cohort | University-hospital | 2390 | 1.42 |
| 1995 | Chiapasco | Italy | Retrospective cohort | University-hospital | 1500 | 1.67 |
| 1997 | Muhonen | Finland | Retrospective cohort | University | 550 | 2.55 |
| 2002 | Berge | Norway | Prospective cohort | University | 1458 | 0.75 |
| 2004 | Benediktsdóttir | Denmark | Prospective cohort | University | 388 | 2.84 |
| 2007 | Blondeau | Canada | Prospective cohort | Private clinic | 550 | 2.18 |
| 2007 | Gbotolorun | Nigeria | Prospective cohort | University-hospital | 331 | 4.23 |
| 2008 | Baqain | Jordan | Prospective cohort | University-hospital | 148  (patients) | 2.03* |
| 2010 | Adeyemo | Nigeria | Retrospective cohort | University-hospital | 506 | 0.20 |
| 2011 | Malkawi | Jordan | Prospective cohort | University-hospital | 327  (patients) | 0.61* |
| 2012 | Christensen | Denmark | Retrospective cohort | University | 114 | 4.39 |
| 2017 | Reiland | USA | Retrospective cohort | Private clinic | 1895 | 2.90 |
| 2020 | Chen | Taiwan | Retrospective cohort | National database | 16609 | 0.17 |
