# Supplementary material for: Revisit incidence of complications after impacted mandibular third molar extraction: A nationwide population-based cohort study
Source: PLoS One. 2021 Feb 22;16(2):e0246625. doi: 10.1371/journal.pone.0246625 (PMC7899344; doi:10.1371/journal.pone.0246625)
Supplement: S3 Table — (DOCX) [file pone.0246625.s003.docx]

S3 Table.

| **Year** | **Author** | **Country** | **Type of study** | **Setting** | **Numbers of iLM3** | **Incidence**  **(%)** |
| --- | --- | --- | --- | --- | --- | --- |
| 1995 | Chiapasco | Italy | Retrospective cohort | University-hospital | 1500 | 0.00 |
| 1995 | de Boer | Netherland | Retrospective cohort | University-hospital | 2390 | 0.04 |
| 2002 | Berge | Norway | Prospective cohort | University | 1035 | 1.74 |
| 2009 | Juhl | Denmark | Prospective match-paired | University-hospital | 72 | 4.17 |
| 2014 | Argrawal | India | Retrospective  cohort | University, hospital (Multi-center) | 270 | 0.74 |
| 2020 | Chen | Taiwan | Retrospective  cohort | National database | 16609 | 0.41 |
